# Supplementary material for: Predictive symptoms for COVID-19 in the community: REACT-1 study of over 1 million people
Source: PLoS Med. 2021 Sep 28;18(9):e1003777. doi: 10.1371/journal.pmed.1003777 (PMC8478234; doi:10.1371/journal.pmed.1003777)
Supplement: S1 Fig — (DOCX) [file pmed.1003777.s001.docx]

**S1 Figure.** Simulation of test allocation and consequent proportions of symptomatic PCR positives (cases) detected, as a function of proportion of all symptomatic people tested, using models trained in rounds 2 to 7 and applied to A) hold-out test data (rounds 2 to 7) and B) round 8. The dotted lines indicate the optimal proportion of testing for symptomatic individuals based on prioritization from the stability selection model. The points linked by dashed lines indicate individuals who did not report any of the seven symptoms positively predictive of COVID-19 from the stability selection model.

**A B**
