# Supplementary material for: Predictive symptoms for COVID-19 in the community: REACT-1 study of over 1 million people
Source: PLoS Med. 2021 Sep 28;18(9):e1003777. doi: 10.1371/journal.pmed.1003777 (PMC8478234; doi:10.1371/journal.pmed.1003777)

**S2 Figure.** Results of age-stratified LASSO stability selection using 1,000 models (with 50% subsamples of training data from rounds 2–7) at A) 5–17, B) 18–54 and C) 55+ years. Positive regression coefficients are presented in blue, and negative in red. Mean (penalized) log Odds Ratio (log OR) across all models are shown in the top panel. Only symptoms selected at least once are displayed. The selection proportion (selection prop.) for each symptom is shown in the middle panel, which is the proportion of 1,000 models that included the symptom; the horizontal dashed line represents the selection threshold of 50%. Symptoms are ordered according to their age-specific selection proportions, and the four classic COVID-19 symptoms are in bold. The bottom panel shows the area under the receiver operating characteristics curve (AUC) of models adding each variable to the model in order of selection proportion (from left to right).


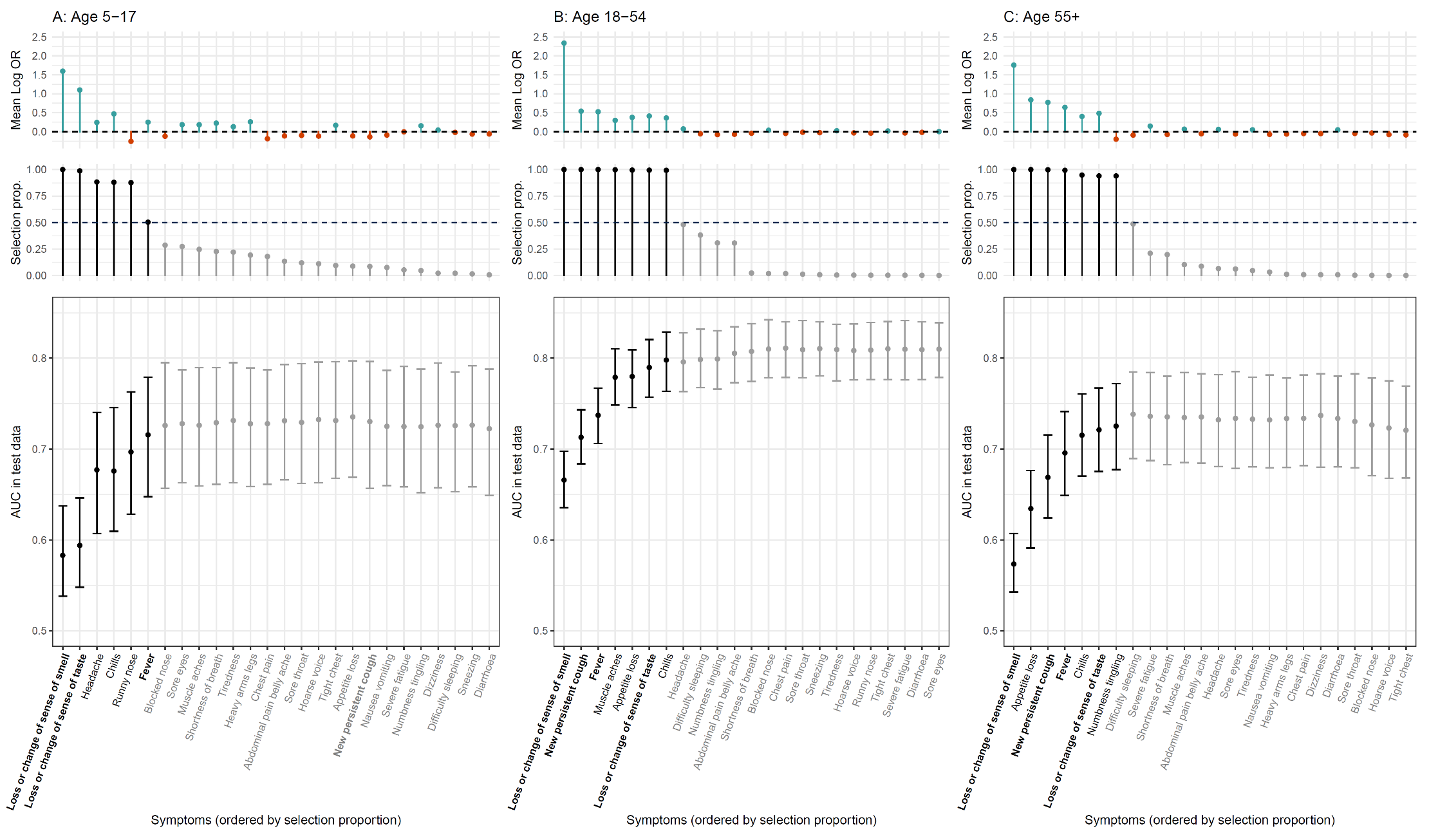

Supplement: S2 Fig — (DOCX) [file pmed.1003777.s002.docx]
