# Supplementary material for: Predictive symptoms for COVID-19 in the community: REACT-1 study of over 1 million people
Source: PLoS Med. 2021 Sep 28;18(9):e1003777. doi: 10.1371/journal.pmed.1003777 (PMC8478234; doi:10.1371/journal.pmed.1003777)
Supplement: S3 Fig — (DOCX) [file pmed.1003777.s003.docx]

**S3 Figure.** Results from univariate logistic regression models of PCR positivity for first reported symptoms. Effect size estimates are expressed as odds ratios (with 95% confidence intervals) in A) rounds 2–7 and B) round 8.

**A** **B**
