# Supplementary material for: Predictive symptoms for COVID-19 in the community: REACT-1 study of over 1 million people
Source: PLoS Med. 2021 Sep 28;18(9):e1003777. doi: 10.1371/journal.pmed.1003777 (PMC8478234; doi:10.1371/journal.pmed.1003777)
Supplement: S4 Fig — (DOCX) [file pmed.1003777.s004.docx]

**S4 Figure.** Results from LASSO stability selection using 1,000 penalized logistic models (with 50% subsamples of training data from rounds 2–7) models on outcome of PCR positivity with first reported symptoms as predictors, applied to holdout test data from rounds 2–7 (grey) and round 8 (red). We report mean log Odds Ratio (log OR), selection proportion (selection prop.) and Area the Under the Curve (AUC) of a logistic model sequentially including symptoms.

**
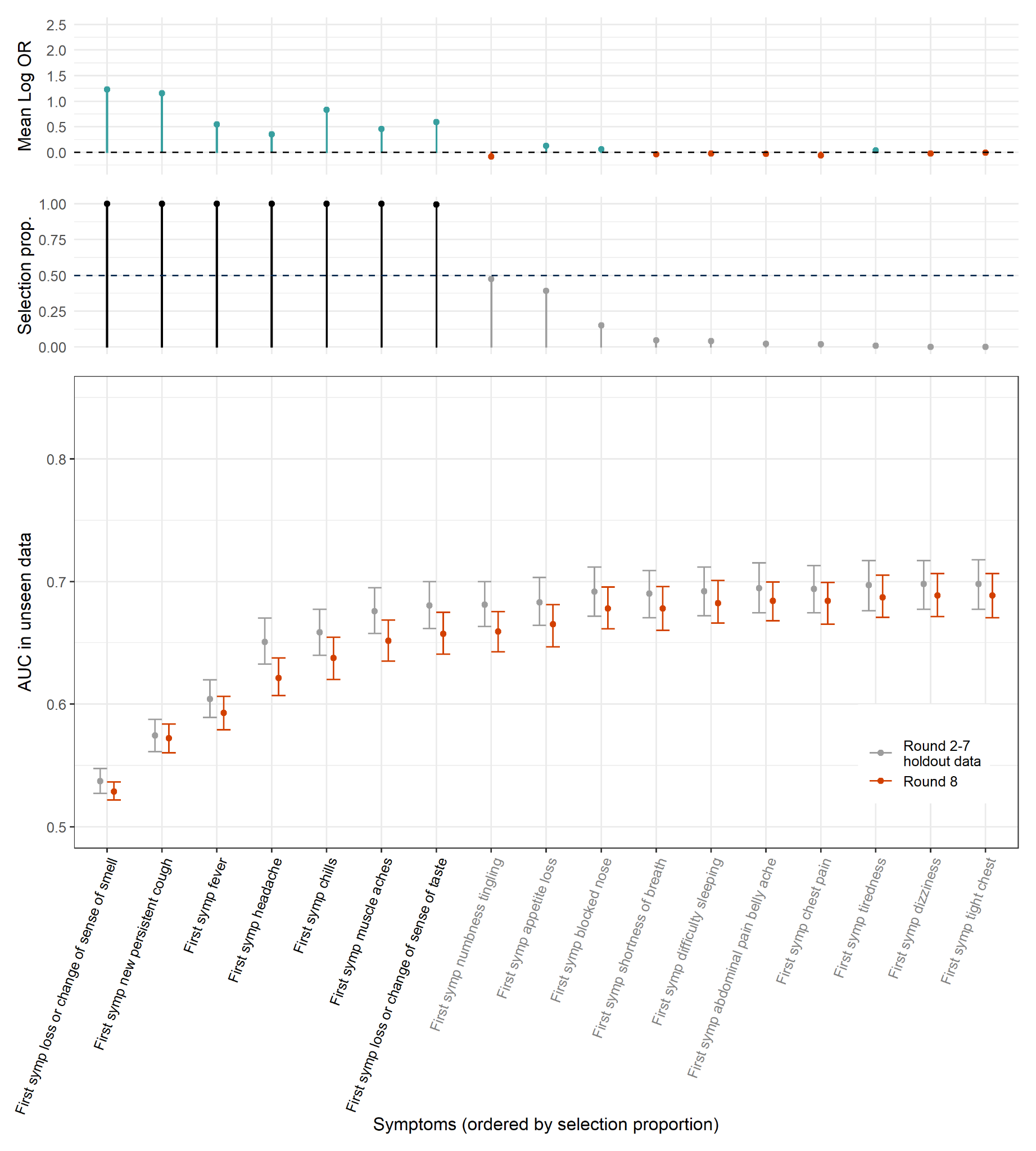
**
