# Supplementary material for: Predictive symptoms for COVID-19 in the community: REACT-1 study of over 1 million people
Source: PLoS Med. 2021 Sep 28;18(9):e1003777. doi: 10.1371/journal.pmed.1003777 (PMC8478234; doi:10.1371/journal.pmed.1003777)
Supplement: S5 Fig — (DOCX) [file pmed.1003777.s005.docx]

**S5 Figure.** Proportion of B.1.1.7 variant among all cases in England, Oct 2020 – February 2021. Estimated proportion of B.1.1.7 variant during the round 6 fieldwork dates was 0.7% [0.6,0.9]. Estimated proportion of B.1.1.7 variant during the round 7 fieldwork dates was 10.6% [10.1,11.2]. Data from Volz et al, *Assessing transmissibility of SARS-CoV-2 lineage B.1.1.7 in England^[[1]](#footnote-1)^*


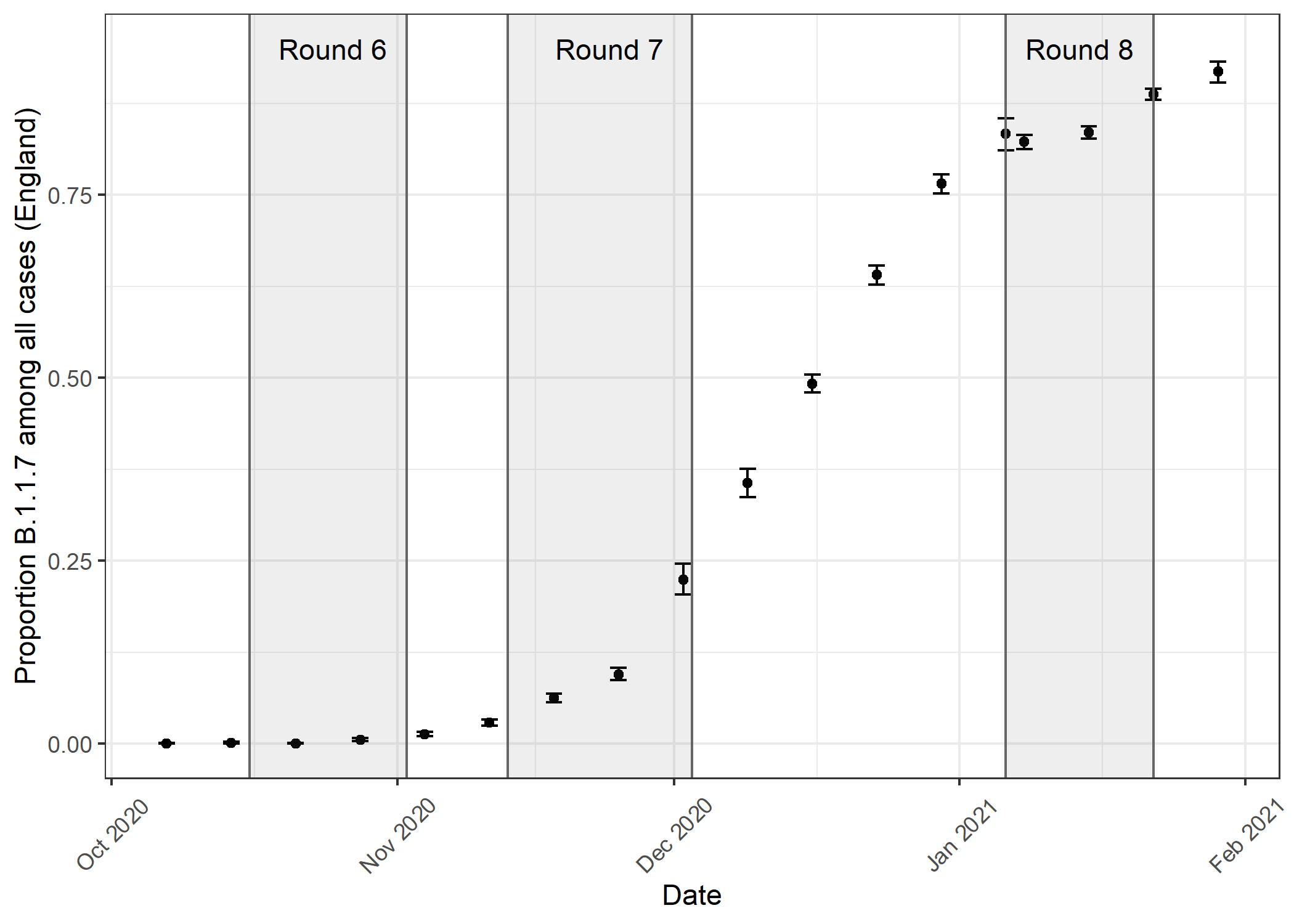


1. Volz, E, Mishra S, Chand M, Barrett JC, Johnson R, Geidelberg L et al. Assessing transmissibility of SARS-CoV-2 lineage B.1.1.7 in England. Nature 593, 266–269 (2021). https://doi.org/10.1038/s41586-021-03470-x [↑](#footnote-ref-1)
