# Supplementary material for: Predictive symptoms for COVID-19 in the community: REACT-1 study of over 1 million people
Source: PLoS Med. 2021 Sep 28;18(9):e1003777. doi: 10.1371/journal.pmed.1003777 (PMC8478234; doi:10.1371/journal.pmed.1003777)
Supplement: S1 Supplementary Methods — (DOCX) [file pmed.1003777.s006.docx]

# Supplementary methods

The analytical plan of the primary outcome of the REACT-1 study (prevalence of the SARS-CoV2 infection in the community) has been published and is available at https://wellcomeopenresearch.org/articles/5-200^[[1]](#footnote-1)^.

Our stability selection LASSO models rely on the approach introduced by Meinhausen and Bühlman (2010) and combine variable selection via L1-penalisation and subsampling to estimate individual feature selection proportions as a proxy for their relevance. In our application, we considered (N=1,000) 50% independent subsamples of the (70%) training data set and fixed the threshold in selection proportion to be 50% (i.e. we considered as stably selected variables those which were included in at least in half of the evaluated models). For each (of the 1,000) subsamples, we fitted a series of LASSO models for an a priori set of 100 values for the penalty parameter (lambda, controlling the sparsity of the model). For each value of the penalty parameter, and based on the a priori fixed value for pi, we calculated the expected per family error rate (PFER) and identified the best penalty value as the smallest value of lambda (i.e. the least sparse possible model) ensuring a control of the PFER below 5. We report the selection proportion of the assayed symptoms for that calibrated value of the penalty parameter and evaluate the predictive performances of the stably selected symptoms (with selection proportion over 50%) in the (30%) testing set by recalibrating a logistic model. Corresponding code is available at <https://github.com/barbarabodinier/Community_detection_COVID-19>.

1. Riley S, Atchison C, Ashby D, Donnelly CA, Barclay W, Cooke GS, Ward H et al. REal-time Assessment of Community Transmission (REACT) of SARS-CoV-2 virus: Study protocol. Wellcome Open Res. 5, 200 (2020). [↑](#footnote-ref-1)
