# Supplementary material for: Predictive symptoms for COVID-19 in the community: REACT-1 study of over 1 million people
Source: PLoS Med. 2021 Sep 28;18(9):e1003777. doi: 10.1371/journal.pmed.1003777 (PMC8478234; doi:10.1371/journal.pmed.1003777)
Supplement: S1 Table — Results are presented for the full study population. (DOCX) [file pmed.1003777.s007.docx]

**S1 Table**: Key characteristics of the REACT-1 study population for rounds 2-7 and round 8 separately. Numbers are reported for the full samples and by test positivity status for the full study population.

|  |  | **Round 2–7** | |  | **Round 8** | | |
| --- | --- | --- | --- | --- | --- | --- | --- |
|  | **Category** | **Test negative** | **Test positive** | **Total** | **Test negative** | **Test positive** | **Total** |
|  | All participants | 975,541 (99.6%) | 4,168 (0.4%) | 979,709 | 165,354 (98.6%) | 2,282 (1.4%) | 167,636 |
| Symptomatic | Not Symptomatic | 868,242 (99.7%) | 2,630 (0.3%) | 870,872 | 145,450 (99.1%) | 1,251 (0.9%) | 146,701 |
|  | Symptomatic | 107,299 (98.6%) | 1,538 (1.4%) | 108,837 | 19,904 (95.1%) | 1,031 (4.9%) | 20,935 |
| Sex | Male | 439,782 (99.6%) | 1,899 (0.4%) | 441,681 | 74,253 (98.6%) | 1,043 (1.4%) | 75,296 |
|  | Female | 535,759 (99.6%) | 2,269 (0.4%) | 538,028 | 91,101 (98.7%) | 1,239 (1.3%) | 92,340 |
| Age | 05-17 | 124,470 (99.6%) | 550 (0.4%) | 125,020 | 21,448 (98.6%) | 314 (1.4%) | 21,762 |
|  | 13-17 | 72,050 (99.5%) | 338 (0.5%) | 72,388 | 11,556 (98.6%) | 170 (1.4%) | 11,726 |
|  | 18-24 | 55,788 (99.3%) | 366 (0.7%) | 56,154 | 8,775 (98.1%) | 172 (1.9%) | 8,947 |
|  | 25-34 | 41,054 (99.2%) | 315 (0.8%) | 41,369 | 6,596 (97.7%) | 156 (2.3%) | 6,752 |
|  | 35-44 | 90,517 (99.5%) | 466 (0.5%) | 90,983 | 14,766 (98.3%) | 263 (1.7%) | 15,029 |
|  | 45-54 | 161,588 (99.5%) | 732 (0.5%) | 162,320 | 27,570 (98.5%) | 412 (1.5%) | 27,982 |
|  | 55-64 | 180,133 (99.6%) | 687 (0.4%) | 180,820 | 31,576 (98.7%) | 413 (1.3%) | 31,989 |
|  | 65+ | 249,941 (99.7%) | 714 (0.3%) | 250,655 | 43,067 (99.1%) | 382 (0.9%) | 43,449 |
| Ethnicity | White | 884,839 (99.6%) | 3,636 (0.4%) | 888,475 | 150,168 (98.7%) | 1,938 (1.3%) | 152,106 |
|  | Asian / Asian British | 41,782 (99.3%) | 275 (0.7%) | 42,057 | 6,374 (97.5%) | 163 (2.5%) | 6,537 |
|  | Black / African / Caribbean / Black British | 11,677 (99.4%) | 66 (0.6%) | 11,743 | 1,771 (96.7%) | 60 (3.3%) | 1,831 |
|  | Mixed | 16,665 (99.6%) | 69 (0.4%) | 16,734 | 2,705 (98.3%) | 46 (1.7%) | 2,751 |
|  | Other | 7,734 (99.4%) | 46 (0.6%) | 7,780 | 1,161 (97.6%) | 29 (2.4%) | 1,190 |
| Region | South East | 219,044 (99.7%) | 650 (0.3%) | 219,694 | 38,574 (98.5%) | 578 (1.5%) | 39,152 |
|  | North East | 35,428 (99.4%) | 214 (0.6%) | 35,642 | 5,669 (98.9%) | 62 (1.1%) | 5,731 |
|  | North West | 110,289 (99.3%) | 783 (0.7%) | 111,072 | 17,697 (98.8%) | 220 (1.2%) | 17,917 |
|  | Yorkshire and The Humber | 64,590 (99.4%) | 399 (0.6%) | 64,989 | 10,572 (99.1%) | 93 (0.9%) | 10,665 |
|  | East Midlands | 124,853 (99.5%) | 588 (0.5%) | 125,441 | 21,472 (99%) | 225 (1%) | 21,697 |
|  | West Midlands | 91,088 (99.5%) | 450 (0.5%) | 91,538 | 14,889 (98.7%) | 198 (1.3%) | 15,087 |
|  | East of England | 142,699 (99.7%) | 396 (0.3%) | 143,095 | 24,787 (98.4%) | 391 (1.6%) | 25,178 |
|  | London | 94,232 (99.6%) | 401 (0.4%) | 94,633 | 15,251 (97.5%) | 390 (2.5%) | 15,641 |
|  | South West | 93,318 (99.7%) | 287 (0.3%) | 93,605 | 16,443 (99.2%) | 125 (0.8%) | 16,568 |
