# Supplementary material for: Predictive symptoms for COVID-19 in the community: REACT-1 study of over 1 million people
Source: PLoS Med. 2021 Sep 28;18(9):e1003777. doi: 10.1371/journal.pmed.1003777 (PMC8478234; doi:10.1371/journal.pmed.1003777)
Supplement: S2 Table — (DOCX) [file pmed.1003777.s008.docx]

**S2 Table.** Symptom prevalence by age group among SARS-CoV-2 PCR test negative and positive respondents in REACT-1 A) rounds 2 to 7 and B) round 8.

## A Rounds 2 to 7

|  | **Age 5-17** |  | **Age 18-54** |  | **Age 55+** |  | **All ages (5+)** |  |
| --- | --- | --- | --- | --- | --- | --- | --- | --- |
| *Symptom* | *Negative* | *Positive* | *Negative* | *Positive* | *Negative* | *Positive* | *Negative* | *Positive* |
| Full cohort | 127838 | 704 | 417631 | 2064 | 430075 | 1401 | 975544 | 4169 |
| Loss of sense of smell | 253 (0.2%) | 40 (5.7%) | 1365 (0.3%) | 309 (15%) | 689 (0.2%) | 90 (6.4%) | 2307 (0.2%) | 439 (10.5%) |
| Loss of sense of taste | 244 (0.2%) | 33 (4.7%) | 1584 (0.4%) | 265 (12.8%) | 937 (0.2%) | 90 (6.4%) | 2765 (0.3%) | 388 (9.3%) |
| New persistent cough | 1148 (0.9%) | 23 (3.3%) | 2958 (0.7%) | 184 (8.9%) | 1492 (0.3%) | 76 (5.4%) | 5598 (0.6%) | 283 (6.8%) |
| Fever | 985 (0.8%) | 41 (5.8%) | 3403 (0.8%) | 195 (9.4%) | 1971 (0.5%) | 98 (7%) | 6359 (0.7%) | 334 (8.0%) |
| Runny nose | 5823 (4.6%) | 68 (9.7%) | 15719 (3.8%) | 266 (12.9%) | 8154 (1.9%) | 123 (8.8%) | 29696 (3.0%) | 457 (11.0%) |
| Sneezing | 4547 (3.6%) | 67 (9.5%) | 15595 (3.7%) | 281 (13.6%) | 8490 (2%) | 130 (9.3%) | 28632 (2.9%) | 478 (11.5%) |
| Blocked nose | 5126 (4%) | 71 (10.1%) | 11954 (2.9%) | 299 (14.5%) | 5465 (1.3%) | 88 (6.3%) | 22545 (2.3%) | 458 (11.0%) |
| Sore eyes | 1083 (0.8%) | 28 (4%) | 7800 (1.9%) | 161 (7.8%) | 6301 (1.5%) | 79 (5.6%) | 15184 (1.6%) | 268 (6.4%) |
| Sore throat | 4792 (3.7%) | 76 (10.8%) | 18702 (4.5%) | 306 (14.8%) | 8184 (1.9%) | 103 (7.4%) | 31678 (3.2%) | 485 (11.6%) |
| Hoarse voice | 1384 (1.1%) | 17 (2.4%) | 4621 (1.1%) | 133 (6.4%) | 3507 (0.8%) | 61 (4.4%) | 9512 (1.0%) | 211 (5.1%) |
| Headache | 4227 (3.3%) | 106 (15.1%) | 26226 (6.3%) | 540 (26.2%) | 12366 (2.9%) | 200 (14.3%) | 42819 (4.4%) | 846 (20.3%) |
| Dizziness | 1042 (0.8%) | 25 (3.6%) | 7502 (1.8%) | 168 (8.1%) | 5118 (1.2%) | 69 (4.9%) | 13662 (1.4%) | 262 (6.3%) |
| Appetite loss | 1193 (0.9%) | 25 (3.6%) | 3887 (0.9%) | 194 (9.4%) | 2481 (0.6%) | 115 (8.2%) | 7561 (0.8%) | 334 (8.0%) |
| Nausea/vomiting | 1267 (1%) | 25 (3.6%) | 5466 (1.3%) | 112 (5.4%) | 2566 (0.6%) | 42 (3%) | 9299 (1.0%) | 179 (4.3%) |
| Diarrhea | 883 (0.7%) | 16 (2.3%) | 6949 (1.7%) | 116 (5.6%) | 3978 (0.9%) | 65 (4.6%) | 11810 (1.2%) | 197 (4.7%) |
| Abdominal pain / belly ache | 2386 (1.9%) | 33 (4.7%) | 8986 (2.2%) | 120 (5.8%) | 5541 (1.3%) | 61 (4.4%) | 16913 (1.7%) | 214 (5.1%) |
| Shortness of breath | 575 (0.4%) | 18 (2.6%) | 6392 (1.5%) | 146 (7.1%) | 6292 (1.5%) | 77 (5.5%) | 13259 (1.4%) | 241 (5.8%) |
| Tight chest | 508 (0.4%) | 16 (2.3%) | 5344 (1.3%) | 146 (7.1%) | 3429 (0.8%) | 58 (4.1%) | 9281 (1.0%) | 220 (5.3%) |
| Chest pain | 387 (0.3%) | 5 (0.7%) | 2582 (0.6%) | 61 (3%) | 1493 (0.3%) | 22 (1.6%) | 4462 (0.5%) | 88 (2.1%) |
| Chills | 904 (0.7%) | 40 (5.7%) | 4199 (1%) | 212 (10.3%) | 2569 (0.6%) | 97 (6.9%) | 7672 (0.8%) | 349 (8.4%) |
| Difficulty sleeping | 1738 (1.4%) | 33 (4.7%) | 15654 (3.7%) | 242 (11.7%) | 10548 (2.5%) | 108 (7.7%) | 27940 (2.9%) | 383 (9.2%) |
| Tiredness | 2884 (2.3%) | 70 (9.9%) | 24240 (5.8%) | 482 (23.4%) | 14930 (3.5%) | 246 (17.6%) | 42054 (4.3%) | 798 (19.1%) |
| Severe fatigue | 271 (0.2%) | 7 (1%) | 2831 (0.7%) | 99 (4.8%) | 1697 (0.4%) | 51 (3.6%) | 4799 (0.5%) | 157 (3.8%) |
| Numbness/tingling | 276 (0.2%) | 7 (1%) | 4917 (1.2%) | 77 (3.7%) | 4721 (1.1%) | 33 (2.4%) | 9914 (1.0%) | 117 (2.8%) |
| Heavy arms/legs | 353 (0.3%) | 13 (1.8%) | 4556 (1.1%) | 136 (6.6%) | 3775 (0.9%) | 61 (4.4%) | 8684 (0.9%) | 210 (5.0%) |
| Muscle aches | 1163 (0.9%) | 34 (4.8%) | 11741 (2.8%) | 378 (18.3%) | 9052 (2.1%) | 172 (12.3%) | 21956 (2.3%) | 584 (14.0%) |

**B** Round 8

|  | **Age 5-17** |  | **Age 18-54** |  | **Age 55+** |  | **All ages (5+)** |  |
| --- | --- | --- | --- | --- | --- | --- | --- | --- |
| *Symptom* | *Negative* | *Positive* | *Negative* | *Positive* | *Negative* | *Positive* | Negative | Positive |
| Full cohort | 20331 | 342 | 70380 | 1145 | 74643 | 795 | 165354 | 2282 |
| Loss of sense of smell | 30 (0.1%) | 19 (5.6%) | 296 (0.4%) | 193 (16.9%) | 161 (0.2%) | 68 (8.6%) | 487 (0.3%) | 280 (12.3%) |
| Loss of sense of taste | 21 (0.1%) | 16 (4.7%) | 317 (0.5%) | 176 (15.4%) | 193 (0.3%) | 83 (10.4%) | 531 (0.3%) | 275 (12.1%) |
| New persistent cough | 39 (0.2%) | 13 (3.8%) | 545 (0.8%) | 167 (14.6%) | 301 (0.4%) | 81 (10.2%) | 885 (0.5%) | 261 (11.4%) |
| Fever | 76 (0.4%) | 9 (2.6%) | 478 (0.7%) | 142 (12.4%) | 312 (0.4%) | 62 (7.8%) | 866 (0.5%) | 213 (9.3%) |
| Runny nose | 320 (1.6%) | 33 (9.6%) | 2997 (4.3%) | 189 (16.5%) | 1917 (2.6%) | 101 (12.7%) | 5234 (3.2%) | 323 (14.2%) |
| Sneezing | 292 (1.4%) | 26 (7.6%) | 2672 (3.8%) | 199 (17.4%) | 1938 (2.6%) | 99 (12.5%) | 4902 (3.0%) | 324 (14.2%) |
| Blocked nose | 330 (1.6%) | 34 (9.9%) | 2160 (3.1%) | 212 (18.5%) | 1230 (1.6%) | 84 (10.6%) | 3720 (2.2%) | 330 (14.5%) |
| Sore eyes | 133 (0.7%) | 19 (5.6%) | 1404 (2%) | 108 (9.4%) | 1075 (1.4%) | 50 (6.3%) | 2612 (1.6%) | 177 (7.8%) |
| Sore throat | 247 (1.2%) | 31 (9.1%) | 2885 (4.1%) | 212 (18.5%) | 1485 (2%) | 77 (9.7%) | 4617 (2.8%) | 320 (14.0%) |
| Hoarse voice | 43 (0.2%) | 7 (2%) | 695 (1%) | 80 (7%) | 567 (0.8%) | 59 (7.4%) | 1305 (0.8%) | 146 (6.4%) |
| Headache | 557 (2.7%) | 50 (14.6%) | 5234 (7.4%) | 345 (30.1%) | 2519 (3.4%) | 158 (19.9%) | 8310 (5.0%) | 553 (24.2%) |
| Dizziness | 150 (0.7%) | 11 (3.2%) | 1307 (1.9%) | 107 (9.3%) | 894 (1.2%) | 64 (8.1%) | 2351 (1.4%) | 182 (8.0%) |
| Appetite loss | 173 (0.9%) | 15 (4.4%) | 713 (1%) | 136 (11.9%) | 473 (0.6%) | 112 (14.1%) | 1359 (0.8%) | 263 (11.5%) |
| Nausea/vomiting | 144 (0.7%) | 7 (2%) | 928 (1.3%) | 70 (6.1%) | 448 (0.6%) | 32 (4%) | 1520 (0.9%) | 109 (4.8%) |
| Diarrhea | 96 (0.5%) | 7 (2%) | 1138 (1.6%) | 85 (7.4%) | 666 (0.9%) | 46 (5.8%) | 1900 (1.1%) | 138 (6.0%) |
| Abdominal pain / belly ache | 292 (1.4%) | 12 (3.5%) | 1550 (2.2%) | 74 (6.5%) | 1009 (1.4%) | 62 (7.8%) | 2851 (1.7%) | 148 (6.5%) |
| Shortness of breath | 76 (0.4%) | 3 (0.9%) | 1287 (1.8%) | 119 (10.4%) | 1159 (1.6%) | 50 (6.3%) | 2522 (1.5%) | 172 (7.5%) |
| Tight chest | 68 (0.3%) | 4 (1.2%) | 1137 (1.6%) | 115 (10%) | 726 (1%) | 45 (5.7%) | 1931 (1.2%) | 164 (7.2%) |
| Chest pain | 37 (0.2%) | 2 (0.6%) | 533 (0.8%) | 54 (4.7%) | 267 (0.4%) | 18 (2.3%) | 837 (0.5%) | 74 (3.2%) |
| Chills | 109 (0.5%) | 11 (3.2%) | 1220 (1.7%) | 165 (14.4%) | 836 (1.1%) | 92 (11.6%) | 2165 (1.3%) | 268 (11.7%) |
| Difficulty sleeping | 301 (1.5%) | 20 (5.8%) | 3341 (4.7%) | 162 (14.1%) | 2193 (2.9%) | 88 (11.1%) | 5835 (3.5%) | 270 (11.8%) |
| Tiredness | 347 (1.7%) | 37 (10.8%) | 4339 (6.2%) | 339 (29.6%) | 2557 (3.4%) | 183 (23%) | 7243 (4.4%) | 559 (24.5%) |
| Severe fatigue | 42 (0.2%) | 6 (1.8%) | 567 (0.8%) | 82 (7.2%) | 313 (0.4%) | 46 (5.8%) | 922 (0.6%) | 134 (5.9%) |
| Numbness/tingling | 28 (0.1%) | 5 (1.5%) | 865 (1.2%) | 44 (3.8%) | 793 (1.1%) | 25 (3.1%) | 1686 (1.0%) | 74 (3.2%) |
| Heavy arms/legs | 41 (0.2%) | 6 (1.8%) | 792 (1.1%) | 98 (8.6%) | 627 (0.8%) | 52 (6.5%) | 1460 (0.9%) | 156 (6.8%) |
| Muscle aches | 112 (0.6%) | 19 (5.6%) | 2163 (3.1%) | 228 (19.9%) | 1536 (2.1%) | 119 (15%) | 3811 (2.3%) | 366 (16.0%) |
