# Supplementary material for: Predictive symptoms for COVID-19 in the community: REACT-1 study of over 1 million people
Source: PLoS Med. 2021 Sep 28;18(9):e1003777. doi: 10.1371/journal.pmed.1003777 (PMC8478234; doi:10.1371/journal.pmed.1003777)
Supplement: S3 Table — Numbers are presented for cases with wild-type (N = 181) and B.1.1.7 (N = 898) infections. The statistical significance of differences between these 2 groups is evaluated using a chi-squared test for categorical variables. We report the p-value for the null hypothesis of no difference. (DOCX) [file pmed.1003777.s009.docx]

**S3 Table**: Characteristics of the round 8 RT-PCR-positive participants in whom lineage data were available. Numbers are presented for cases with wild (N=181) and B.1.1.7 (N=898) infections. The statistical significance of the differences between these two groups is evaluated using a Student’s T-test for continuous variables and a chi-square test for categorical variables. We report the p-value for the null hypothesis of no differences.

|  | **Category** | **B.1.1.7** | **Wild type** | **Total** | **p-value** |
| --- | --- | --- | --- | --- | --- |
|  | All participants | 898 | 181 | 1,079 |  |
| Sex | Male | 420 (46.8%) | 87 (48.1%) | 507 | 0.813 |
|  | Female | 478 (53.2%) | 94 (51.9%) | 572 |  |
| Age | 5-17 | 55 (6.1%) | 13 (7.2%) | 68 | 0.066 |
|  | 13-17 | 33 (3.7%) | 8 (4.4%) | 41 |  |
|  | 18-24 | 68 (7.6%) | 11 (6.1%) | 79 |  |
|  | 25-34 | 116 (12.9%) | 14 (7.7%) | 130 |  |
|  | 35-44 | 131 (14.6%) | 23 (12.7%) | 154 |  |
|  | 45-54 | 180 (20%) | 29 (16%) | 209 |  |
|  | 55-64 | 169 (18.8%) | 37 (20.4%) | 206 |  |
|  | 65+ | 146 (16.3%) | 46 (25.4%) | 192 |  |
| Ethnicity | White | 768 (87.3%) | 157 (88.2%) | 925 | 0.963 |
|  | Asian / Asian British | 62 (7%) | 12 (6.7%) | 74 |  |
|  | Black / African / Caribbean / Black British | 22 (2.5%) | 4 (2.2%) | 26 |  |
|  | Mixed | 12 (1.4%) | 3 (1.7%) | 15 |  |
|  | Other | 16 (1.8%) | 2 (1.1%) | 18 |  |
| Region | South East | 240 (26.7%) | 24 (13.3%) | 264 | 4.80E-12 |
|  | North East | 17 (1.9%) | 10 (5.5%) | 27 |  |
|  | North West | 84 (9.4%) | 31 (17.1%) | 115 |  |
|  | Yorkshire and The Humber | 25 (2.8%) | 19 (10.5%) | 44 |  |
|  | East Midlands | 92 (10.2%) | 26 (14.4%) | 118 |  |
|  | West Midlands | 78 (8.7%) | 25 (13.8%) | 103 |  |
|  | East of England | 148 (16.5%) | 23 (12.7%) | 171 |  |
|  | London | 171 (19%) | 13 (7.2%) | 184 |  |
|  | South West | 43 (4.8%) | 10 (5.5%) | 53 |  |
