# Supplementary material for: Predictive symptoms for COVID-19 in the community: REACT-1 study of over 1 million people
Source: PLoS Med. 2021 Sep 28;18(9):e1003777. doi: 10.1371/journal.pmed.1003777 (PMC8478234; doi:10.1371/journal.pmed.1003777)
Supplement: Attachment — Submitted filename: Response_to_reviewers_final.docx [file pmed.1003777.s010.docx]

**Requests from the editors:**

1. *Please update the final sentence of the ‘Methods and Findings’ section of your abstract to list 2-3 of the study’s main limitations (the sentence should begin ‘Limitations of this study include…’ or similar).*

We have listed the two main limitations of our study in the abstract, as requested (see p.3 l.65-70).

2. Please ensure you have provided all the necessary information that would allow a reader to access the data used in this study (for example, contact information for the project steering committee) in your response to the relevant question in the submission form. See <https://journals.plos.org/plosmedicine/s/data-availability>for more information.
   As stated in the Data Sharing Statement, **:** Summary statistics and descriptive tables are available in Supplementary information; R scripts used to perform the analyses can be downloaded at <https://github.com/barbarabodinier/Community_detection_COVID-19>
3. If the study has an associated protocol and/or analysis plan, please can these be provided as supplementary materials.
   The REACT study design has been detailed in a previous publication (citation number 7). It includes the study protocol and the analytical plan for the primary outcome of the study: the prevalence of SARS-CoV2 infection. The present work analyses the symptomatology of COVID-19 PCR positivity and therefore focuses on secondary outcomes of the REACT study. The questionnaires used are on the study website (https://www.imperial.ac.uk/medicine/research-and-impact/groups/react-study/react-1-study-materials/). For these secondary analyses we developed an analytical plan as detailed in our response to statistical reviewer point 7, and which is now included in Supplementary methods together with the link to accompanying R-scripts.
4. Please add a completed STROBE checklist as a supplementary file, labelled "S1_STROBE_Checklist" or similar and referred to as such in the Methods section.
   In the checklist, please refer to individual items by section (e.g., "Methods") and paragraph number, not by line or page numbers as these generally change in the event of publication.
   The STOBE checklist has now been added to the present revision.
5. Please update your in-text citations to appear before punctuation.
   In-text citations were updated accordingly.
6. The final paragraph of the ‘Introduction’ can be shortened to state the study’s aims only; elements of discussion can be relocated to the ‘Discussion’ section.
   We have reformulated the last sentence of the introduction, so it only mentions the study aims (see p.4, l90-01).
7. Please remove the ‘Transparency’ and ‘Conflict of Interest’ statements from the end of the main text. The relevant information should be captured in your responses to the submission forms and, in the event of publication, will be presented as metadata.

These two statements have been removed from the main text.

**Comments from the reviewers:**

**Reviewer #1:** Thanks for the opportunity to review your manuscript. My role is as a statistical reviewer, so my comments and questions focus on the study design, data, and analysis presented in the manuscript. I have put general questions first, and then followed these with queries specific to a section of the manuscript (with a line/page reference).

This manuscript presents a study designed to identify key symptoms of COVID-19. This data is from community prevalence surveys in the UK where participants are recruited, questioned about symptom history and have a swab tested for COVID-19. The symptoms were identified by splitting surveys 2-7 into training and test data, and variable selection was achieved by using LASSO logistic regression with all the recorded symptoms. Within the test data sets, a resampling approach was used to estimate a stability of selection parameter, with variables selected 50% of the time were selected. This model was validated against the test set from surveys 2-7 (same temporality) and in survey 8. This is a complex but robust approach to variable selection with both LASSO and the resampling within training data. There was a fairly good level of performance in the round 8 data (AUC=0.77) given that the symptoms are all self-report.

8. There are some new approaches used here that I am not familiar with (i.e. particularly the approach to stability selection penalty parameter), is the code used for the analysis able to be shared? This would help me understand what you did and be able to fully recommend it.

   As pointed out by the reviewer our analyses included stability selection approaches for (LASSO) penalised regression models, as proposed by Meinhausen and Buhlman (Reference number 12). In this approach the variable selection from the L1-penalised regression is combined with subsampling of the population to estimate the selection proportion of individual features across the ensemble of LASSO models. This introduces a second parameter (in addition to the penalty) pi, the threshold in selection proportion above which features are considered stable. In our application, we considered (N=1,000) 50% subsamples of the (70%) training set. We fixed the threshold in selection proportion to be 50% (i.e. we considered as stably selected variables those which were included in at least in half of the evaluated models). For each (of the 1,000) subsamples, we fitted a series of LASSO models for an a priori set of 100 values for the penalty parameter (lambda, controlling the sparsity of the model). For a given value of lambda, and based on the a priori fixed value for pi, we calculated the expected per family error rate (PFER) and identified the best penalty value as the smallest value of lambda (i.e. the least sparse possible model) ensuring a control of the PFER below 5. These details have now been included in the Supplementary Methods, and corresponding code is available at <https://github.com/barbarabodinier/Community_detection_COVID-19>.
9. A pre-registered study protocol is available online for the overall REACT study.  Was an analysis plan for this study created, and if so, is this available for the review?

The study protocol for the main outcome and corresponding analysis (prevalence of SARS-CoV2 infection in all lower-tier local authority areas in England) has been published (citation number 7). The present analyses focuses on reported symptoms, which are secondary outcomes of the study. The questionnaires used are on the study website (https://www.imperial.ac.uk/medicine/research-and-impact/groups/react-study/react-1-study-materials/). For these secondary analyses we developed an analytical plan (see above) included in Supplementary methods together with the link to accompanying R-scripts.

10. I have checked but I couldn't find what the overall recruitment rate for the REACT-1 study was. It looks as though the study uses quota sampling to meet the sample size objectives, how many potential participants at each wave were approached but didn't respond, relative to those that did participant in the study? (see question below about survey design and generalisability)

The response rates are detailed in the table below (also available at <https://www.imperial.ac.uk/media/imperial-college/institute-of-global-health-innovation/REACT1-R12---REACT2-R6---Fieldwork-info-table.pdf>). It is not a quota sample, but the numbers of invitations sent out are designed to obtain a similar number of responses in each local (lower-tier local authority area [LTLA]) area in England; since LTLA populations are different sizes, the proportion of the population invited varies between them. Invitations were sent to a stratified (by LTLA) random sample of named individuals aged 5 years and above obtained from the National Health Service register of patients in England (nearly everyone in England is included on this register). There was no substitution if people did not respond.

11. P7, L131. What software (R?) and packages were used to complete the analysis?

All calculations were done using R version 4.0.2, using the logistic-LASSO algorithm as implemented in the glmnet package and in-house scripts for the stability selection (available at https://github.com/barbarabodinier/Community_detection_COVID-19). This information has now been added at the end of the Methods section (see p 7 l.181-184)

12. P7, L132. To clarify, the main analysis was repeated by COVID strain, age groups, and using first reported symptoms with each sub-group repeated separately from the others?

The reviewer is right, we implemented a series of three sensitivity analyses:

a. Investigating if the symptoms reported to predict test positivity were consistent across age groups
b. Exploring if/which symptoms could discriminate those infected by the wild vs the B.1.1.7 strain in test positives
c. Restricting the list of predictive symptoms to the first reported ones (rather than any reported symptoms in the week before the test).

This has now been clarified in the Methods section (see p7 l.174-180)

13. P11, L235. Do you mean literally mean a 'predictive' model be applied in other contexts with the 7 symptoms or that policy around access to testing, isolation etc. should expand to include the extra symptoms?

Thanks for pointing this out. We meant that the extended list of symptoms we identified should be considered in the test allocation and isolation policy implementation. This has now been clarified in the Discussion (see p.12 l.310-313)

14. P11, L259. This is a good point - is there any data available from the overall survey that can show that this was achieved? I tried looking but couldn't find any.

The sampling and study design have been detailed in the study protocol (citation number 7). For clarity, we have added a summary table in the supplementary materials describing the study population (see Supplementary Table 1).

15. P11, L263. 'Inaccurate' is very broad - what is a likely form of recall bias, and would induce spurious associations, or conceal real associations with this methodology? I have seen 'negative control' type of questions added to surveys e.g. symptoms that could not be associated with the disease included to see if there is an association with the outcome (although this might be hard given the range of symptoms COVID seems to bring about).

For clarity, and as suggested we have reworded the sentence so it reads (see p14, l380-385): ‘These may be subject to recall bias and may not precisely represent the individual dynamics of symptoms onset. However, our self-reported data share the same limitations and therefore accurately mimic the general population symptoms data on which test allocation and isolation measures are based. In addition, participants were unaware of their test results at the time of symptom report which would limit reporting and information bias.’

**Reviewer #2:** This is an excellent study, methodologically well done, well written, and of public health importance, which leaves me little to say.

I have only a few suggestions:

16. (1) B117 was spreading in England from Sep 2020, and so would have been present in Round 5-7 samples. This confounds the comparison of Round 8 with earlier rounds, so please provide an estimate of what percentage of infections in England were likely B117 in earlier months, based on existing public domain estimates. It would have been interesting to show an additional analysis comparing Rounds 2-4 vs Round 8 for similar reasons, but this is not essential.

The first case of B.1.1.7 infection in England was reported in mid-September 2020 and we agree rounds 5 to 7 may potentially include B.1.1.7 cases. Estimated proportions of B.1.1.7 variant infection among all SARS-CoV2 infections in England are 0.7% [0.6,0.9], and 10.6% [10.1,11.2] for November (round 6) and December (round 7), respectively (per data from Volz *et al*; see supplementary figure 5)^[1]^. While it is true that some B.1.1.7 infections may contribute to the overall round 2–7 population these would only represent 3–4% of the total number of cases in rounds 2–7. Additionally, our analyses comparing cases of wild vs B.1.1.7 infection using lineage data in round 8 are suggestive of a consistent symptomatology across both types of infections. This suggests that the possible confounding by B.1.1.7 incidence in rounds 5–7 is likely to have limited impact on the symptoms found predictive of PCR positivity. Models based on rounds 2–4 would be free of such possible confounding but during this period only 60 PCR-positive cases were recorded. Despite this very limited sample size, models trained on 80% of these (N=40) positive cases, identified six of our seven symptoms (ie all except muscle aches) within in the nine most stably selected symptoms.

We have included this point as a limitation of the study in Discussion and included the estimates of B.1.1.7. by round in that discussion (see p15 l404-410).

17. (2) P11/L234-236
    This is a very important point, which perhaps could be stated more clearly for the reader. The important message here is that in countries where testing is being rationed by being based only on the first four symptoms, then almost certainly increasing the symptom list by the other 3 symptoms will be almost as efficient, whilst identifying a significantly larger number of infections.

As detailed in the response to point 12 from reviewer 1 we have clarified and emphasised our statement (see p.12 l.310-315; and p12 l.316-322).

18. (3) P12/L238-239
    It's not clear to me what the intention was of making the reference to economically disadvantaging individuals. The issue of economic disadvantage at the individual level applies equally however restrictive the testing regime, since almost everyone who tests positive will be infected. I think the authors are trying to convey an economic point at the level of the population. But from an economic perspective, the best way to minimize the economic disadvantage of isolation must be to reduce the number of infected persons in the population, which is best achieved by reducing transmission (Ref) as much as possible. To achieve this, isolating a greater fraction of infected cases is always better, so increasing the number of individuals so identified in the short-term will always reduce the net number of people needing isolation in the longer-term.

Our point was that the economic consequences of self-isolation in socially disadvantaged populations may lead in the short-term to a decreased adherence to such preventive measures. Failing to implement an efficient support policy targeting vulnerable populations will induce a social gradient in the prevention and in turn in the susceptibility to COVID-19 infection. We have hopefully clarified this in the Discussion. We agree with the reviewer that short-term measures and their longer-term consequences should be balanced to achieve a sustainable drop in transmission. These points are now discussed together with the points raised in point 18 below (see pp.12-13 l.322-347)

(4) I refer to the following papers:

Hellewell J, Abbott S, Gimma A, Bosse NI, Jarvis CI, Russell TW, et al. Feasibility of controlling COVID-19 outbreaks by isolation of cases and contacts. Lancet Glob Health. 2020;8(4):e488-e96.
Kerr et al, 2021, Nature Communications. Controlling COVID-19 via test-trace-quarantine. <https://www.nature.com/articles/s41467-021-23276-9>

Thank you for bringing these two publications to our attention. These were added to the reference list.

19. I think efficiency is important in a context where testing capacity is limited, so this paper helps inform the testing policy in those contexts. However, to successfully control COVID-19, efficiency is not the only criterion. Effectiveness is also important, ie maximization of the fraction of all infections detected is critical to any strategy that aims to reduce R below 1, especially one that seeks to avoid damaging social mobility restrictions. I would suggest the authors discuss the implications for the less constrained settings, and scenarios where the primary goal is to stop transmission.

    Specifically, the authors should discuss further their finding that the 7 symptoms would still only identify ~70% of symptomatic individuals, or 30-40% of all infected cases (after accounting for asymptomatic infections. Given that a large fraction of those with the relevant symptoms will not voluntarily test in the most ideal circumstances, what this means is that in practice the actual percentage of symptomatic cases that will be tested and detected will be far less. What the findings demonstrate is that the kind of strategy that Australia and New Zealand pursued makes a lot of sense. They used the full list of 26 symptoms to trigger testing (eg <https://www.nsw.gov.au/covid-19/health-and-wellbeing/symptoms-and-testing>). This does result in a much lower positivity rate than would be achieved by restricting to the more efficient subset of 7 symptoms (<0.1% vs 1%), but it achieves much better control of transmission (sufficient to achieve short-term elimination), and in the
    long run required less overall testing than in the UK (2 per 1000/d vs 20 per 1000/day). So for balance, the issue of (cost) efficiency needs to be considered in both the short term and long-term perspectives.

    We thank the reviewer for this interesting point and we have now added a paragraph in the Discussion describing the balance between short-term efficiency and longer term alternatives (see p12-13; l316-339).

**Reviewer #3:** This study seeks to answer a policy-important question in a large representative population. This is a prevalence study using repeated cross-sectional surveys in a representative sample of the community in England aiming to identify a set of symptoms to predict SARS-CoV-2 PCR positivity and distinguish between wild type and B.1.1.7 strains. The surveys consisted of self-administered nose / throat swabs and questionnaires to a randomly selected sample. Significant symptoms were identified over 6 rounds of testing between June and December 2020 and were validated in a sub-population from these first 6 rounds and in an additional 7th round of testing in January 2021 when the B.1.1.7 strain was predominantly circulating. A large population of over 1 million people over the age of 5 were included. Seven symptoms were identified, and the authors have concluded that these may be more efficient than the current four symptoms recommended for testing eligibility within
the UK national testing programme at present. This is one of the largest studies of this type that has been conducted and has monitored changes over the course of the pandemic.

Major issues

20. 1.	It would be useful to have a table or description of the included population (eg age, sex, region, occupations, socio-economic status, number of households) and number of participants per round of testing.

We have added a summary table describing the characteristics of the study population for rounds 2-7 and 8 separately (see Supplementary Table 1).

Minor Issues

21. 2.1	At what time point were surveys administered - before or after the swab? Did they relate to just the day that the swab was administered or to a period of time before sampling?

The surveys were self-administered at the same time as the swab: participants received their kits, were asked to complete the survey, and the swab was collected for analysis. At the time of survey completion, the participants were unaware of the results of their swab test. This has been clarified in the Methods section (see p. 5 l110-121)

22. 2.2	Were baseline questionnaires administered to allow chronic symptoms to be distinguished from acute?

Participants were asked about symptoms that may have emerged in the week before taking the test. These should therefore only capture incident symptoms. Participants were also asked about the duration of their symptoms (see questionnaires website https://www.imperial.ac.uk/medicine/research-and-impact/groups/react-study/react-1-study-materials/). They were in addition asked about a range of pre-existing conditions which would give an indication of chronic illness, and we only considered incident symptoms in the present analyses.

23. 2.3	How were the symptoms that were included in the questionnaire selected?

As detailed in the Methods section the list of 26 symptoms assayed included those suggested previously to be related to SARS-CoV-2 infection: coryzal, gastro-intestinal, cardio-respiratory, and fatigue-related symptoms as well as other flu-like symptoms. This list is consistent with the symptoms for clinical evaluation of the infection and/or severity of patients presenting with suspected COVID-19.

24. 2.4	Were all swabs and viral isolates processed in the same laboratory? If not, is there a difference in assay performance that could affect the results?

Upon completion swabs were sent via courier for RT-PCR testing to one laboratory which has been used in every round of data collection throughout. Samples were analysed according to a standardised protocol. Samples (dry swabs) were refrigerated by the participant and then picked up by courier and maintained at cold temperature until reaching the lab, to minimise any effects of collection/transport on the results.

25. 2.5	There is a potential for selection bias due to survey response. Were there any differences between survey responders and non-responders?

There are limited data available on non-responders, but we have done some (unpublished) analysis showing response rates by age, sex, deprivation and ethnicity and lower-tier local authority (LTLA). In common with most surveys, males, people younger or older than those aged 55-74 years, people in deprived areas, minority ethnic groups and people in urban LTLAs are less likely to respond. We addressed the variable response through sample weighting, although we acknowledge the potential for selection bias as some of these factors are associated with probability of infection, and may be associated with symptom reporting. We have added this point to the limitations section in Discussion (see p 14-15 l.389-404).

26. 2.6	There is no mention of whether sampling was carried out with or without replacement, although the study protocol states that it was with replacement. How were repeat samples in the same individuals accounted for?

Participant sampling has been carried out in order to achieve approximately equal numbers of participants in each LTLA. Each sample in each LTLA was randomly selected, but larger numbers were selected in some LTLAs to address variable response rates at LTLA level. The aim was to achieve a separate (non-overlapping) sample at each round. We therefore approached a different base sample at each round and so we did not have repeat samples to account for. We have now further clarified the sampling approach in Methods (p5 l110-121).

27. 2.7	Please describe any exclusion criteria if these were present.

The REACT-1 sample had no exclusions other than age (5 years and above). For the purpose of this report we analysed data for those with available data on symptom status and with a valid PCR test. An additional 25 participants were excluded due to missing information on sex (see p6 l148-149).

28. 2.8	Please describe the sample size calculation.

Sample size calculations of the REACT-1 study were conducted in relation to the primary outcome of the study: the SARS-CoV-2 infection prevalence in the community. The ~150,000 participants included per round was estimated to ensure a minimal detectable prevalence of the infection of 1.2% in each LTLA with level of confidence of 95%, assuming a conservative 65% diagnostic sensitivity of the PCR test.

29. 2.9	When comparing the wild type and the B.1.1.7 groups - were there any systematic differences (eg age) that could have introduced bias?

As indicated in Supplementary Table 1, there are only small differences in the characteristics of participants from round 2-7 and round 8, irrespective of test positivity. As suggested, we also compared the characteristics of the (N=898) B.1.1.7 PCR positive cases and of the (N=181) wild type PCR positive cases in round 8 (Supplementary Table 3), and found that only the region were different (marked excess of B.1.1.7 cases in East of England, South East and in London). This has now been added to the results section (p10 l257-259).

30. 2.10	In discussion section, it would be useful to mention whether there have been other studies that have identified symptoms that can predict PCR positivity / developed risk scores and how this study compares to them.

We have now referred to other studies looking at predictive symptoms of PCR positivity (see p13 l348-356).

1. Volz et al. Assessing transmissibility of SARS-CoV-2 lineage B.1.1.7 in England. 2021 *Nature* **593**, 266–269. https://doi.org/10.1038/s41586-021-03470-x
