# Supplementary material for: Predictive symptoms for COVID-19 in the community: REACT-1 study of over 1 million people
Source: PLoS Med. 2021 Sep 28;18(9):e1003777. doi: 10.1371/journal.pmed.1003777 (PMC8478234; doi:10.1371/journal.pmed.1003777)
Supplement: Attachment — Submitted filename: Response_to_comments.docx [file pmed.1003777.s011.docx]

**Requests from Editors:**

1. At this stage, we ask that you include a short, non-technical Author Summary of your research to make findings accessible to a wide audience that includes both scientists and non-scientists. The Author Summary should immediately follow the Abstract in your revised manuscript. This text is subject to editorial change and should be distinct from the scientific abstract. https://journals.plos.org/plosmedicine/s/revising-your-manuscript#loc-author-summary
   We have now added an author summary in the manuscript (see p4)
2. Please add further detail to your data availability statement regarding how a reader could apply for access to the REACT study data via the project steering committee (a website or email address ought to be sufficient).

The email address of the two corresponding authors to whom access to REACT1 summary statistics data should be addressed have been added to the statement.

3. Please reference the study questionnaires and the address at which they are located in your Methods section.

The link to the questionnaire onto the study website have been added to the methods section (see p. 6 l140-141).

4. Please remove the ‘Contributors’, ‘Funding’, and ‘Data sharing statement’ from the end of the main text. In the event of publication, this information will be published as metadata based on your responses to the submission form.

These sections have been removed from the main text.

5. Please relocate the ‘Patient and public involvement’ and ‘Dissemination to participants and related patient and public communities’ sections to the Methods, rather than placing them at the end of the main text.

These sections have been moved to the methods.

6. Citations should be in square brackets, not in superscript, and preceding punctuation.

Please use the "Vancouver" style for reference formatting, and see our website for other reference guidelines <https://journals.plos.org/plosmedicine/s/submission-guidelines#loc-references>
After updating your references to use Vancouver style, please ensure that references do not have periods after initials, use ‘et al.’ only after listing the first six authors, and do not contain any formatted text (e.g., bold, italics).

Citations have been reformatted accordingly.

7. Please define any abbreviations used in figures (e.g., OR in Figures 3 and 4).
   The selection proportion (Selection prop.), Area Under the Curve (AUC), and Odds Ratios (OR) used in figures 3-5 have been explicitly defined in the figure legend.

   **Comments from Reviewers:**

**Reviewer #1: Thank you for the revised manuscript and responses to my original queries.** 

8. The additional explanation and available code for the Meinhausen and Buhlman stability selection approach were helpful thank you, I have also looked at the original reference and agree with you this is an appropriate approach.

The rest of the clarifications to the manuscript address my original queries - the part of the discussion about generalisability and potential for bias looks better now. I agree with the comment about timing of self-report and availability of test results, and given the short time period between recall of symptoms and test I think the potential for bias here is limited.
We thank the reviewer for this positive feedback.

9. My only query was about the analysis plan - on p2 of the sup information document there is the extra explanation of the stability selection approach and the link to the code, but I could not see an analysis plan or link to one. Have I inadvertently missed this? This could added to the supplementary appendix easily.

The original analytical plan for the primary outcome: the prevalence of the SARS-CoV2 infection in the community is detailed in citation 9. The link to the corresponding paper has now been added to the supplementary methods.

**Reviewer #2: Thank you for the revisions which address the comments by me and other reviewers.**

10. The only comment I would make is that in the last paragraph of the discussion, the use of the term "most efficient" is incorrect. Technically speaking, using the first ranked symptom would be the most efficient. What I think you mean is that the combination of seven symptoms identified would be the most optimal given the need to maximise case detection in the community under the constraints to testing capacity that existed in conditions similar to that in England in the relevant time period.

We agree with the reviewer, and we have reworded the sentence accordingly. It now reads ‘In summary, we show that a combination of seven symptoms would maximize the case detection rate in the community under testing capacity constraints such as those faced in England between June 2020 and January 2021’ (see p18 l-397-399)
