# Supplementary material for: Predictive symptoms for COVID-19 in the community: REACT-1 study of over 1 million people
Source: PLoS Med. 2021 Sep 28;18(9):e1003777. doi: 10.1371/journal.pmed.1003777 (PMC8478234; doi:10.1371/journal.pmed.1003777)
Supplement: Attachment — Submitted filename: Response_to_comments.docx [file pmed.1003777.s012.docx]

**Requests from Editors:**

1. The data availability statement has changed since the last revision but not as described in your rebuttal letter – the previous revision read ‘Summary statistics and descriptive tables are available in Supplementary information; R scripts used to perform the analyses can be downloaded at <https://github.com/barbarabodinier/Community_detection_COVID-19>. REACT data can be accessed upon application and acceptance to the project steering committee.’ In the latest revision, the statement reads ‘Summary statistics and descriptive tables from REACT-1 study are available on the study website <https://www.imperial.ac.uk/medicine/research-and-impact/groups/reactstudy/react-1-study-materials/> and, together with the R scripts used to perform the analyses, at <https://github.com/barbarabodinier/Community_detection_COVID’>.

My issues with this are as follows:
* The summary statistics and descriptive tables are in the supplementary materials; the web address you have provided contains the questionnaires used. Please correct the statement to include both.
* It looks as though the statement has been truncated or text is missing, as ‘and, together’ does not scan properly and the github address is now missing ‘-19’ and leads to an error page. Please check carefully.
* The email addresses you mention having added in your rebuttal later are not available (in fact reference to the project steering committee has been removed altogether). Please check and see the comment below.
* Finally (and importantly), a study author cannot be the contact person for the data – if there is a contact within the project steering committee that is not a named author, or the option to use a data access or ethics committee, please provide their details. If you foresee this being a problem, please contact me ([cdavidson@plos.org](mailto:cdavidson@plos.org)) to discuss.

We apologise if the different versions of data accessibility statement generated confusion. As detailed in our correspondence to the editor, we are only authorised to share summary statistics and descriptive tables on the REACT-1 data.

These are in SI and will be accessible on the GitHub page (in Excel format), where codes are also available.

We have also added in the statement a link to (i) the study materials (including questionnaires) and (ii) rounds reports where details of the study as well as other data descriptive can be accessed.

We amended the Data Access statement as follows (and double checked the accuracy of the links):

‘Access to REACT-1 data is restricted due to ethical and security considerations. Summary statistics and descriptive tables from the current REACT-1 study are available in the Supplementary Information and at the following link https://github.com/mrc-ide/reactidd/tree/master/inst/extdata/symptoms_prediction_paper

Source codes used for the present analyses are available at https://github.com/mrc-ide/reactidd/tree/master/R/symptom_prediction_paper_scripts

Additional summary statistics and results from the REACT-1 programme are also available at:

<https://www.imperial.ac.uk/medicine/research-and-impact/groups/react-study/real-time-assessment-of-community-transmission-findings/>

and

<https://github.com/mrc-ide/reactidd/tree/master/inst/extdata>

REACT-1 Study Materials are available for each round at <https://www.imperial.ac.uk/medicine/research-and-impact/groups/react-study/react-1-study-materials/> ’

Line 80: Please update this bullet point to ‘Data were collected from over 1 million participants in the REACT-1 study (June 2020 to January 2021), from whom 26 symptoms were assayed and the results of a PCR test were available.

Text has been modified accordingly

Line 83: Please update this bullet point to ‘Adopting a variable selection approach, we sought to determine the best combination of symptoms jointly and complementarily predictive of PCR positivity and compared whether these symptoms were the same between individuals infected by the wild-type virus and those infected by the B.1.1.7 variant’. The following bullet point (line 86) can then be removed.

We have merged the two bullet points as suggested and modified the text as proposed.

Line 89: Please update this bullet point to ‘We identify seven symptoms that were jointly predictive of PCR positivity and appeared to vary only marginally across age groups: loss or change of sense of smell, loss or change of sense of taste, fever, new persistent cough, chills, appetite loss and muscle aches’. The following bullet point (line 92) can then be removed.
Text has been changed accordingly.
Line 99: Please update ‘but will enable’ to ‘but would enable’ 
Text has been changed accordingly.

Line 455: Please remove the periods after the author initials in reference 15.

We removed the periods after the initials in the reference list.
